# Landscape features influencing gene flow and connectivity of an endangered passerine

**DOI:** 10.1002/ece3.11078

**Published:** 2024-05-16

**Authors:** Daniel Bustillo‐de la Rosa, Adrián Barrero, Juan Traba, Jesús T. García, Manuel B. Morales, Ella Vázquez‐Domínguez

**Affiliations:** ^1^ Terrestrial Ecology Group (TEG‐UAM). Department of Ecology Universidad Autónoma de Madrid Madrid Spain; ^2^ Centro de Investigación en Biodiversidad y Cambio Global (CIBC‐UAM) Universidad Autónoma de Madrid Madrid Spain; ^3^ Instituto de Investigación en Recursos Cinegéticos (IREC, CSIC‐UCLM) Ciudad Real Spain; ^4^ Departamento de Ecología de la Biodiversidad, Instituto de Ecología Universidad Nacional Autónoma de México Ciudad de México Mexico

**Keywords:** *Chersophilus duponti* subsp. *duponti*, Dupont's lark, functional connectivity, landscape genetics, resistance surfaces, shrub steppes

## Abstract

Dispersal of individuals and gene flow are crucial aspects to maintain genetic diversity and viability of populations, especially in the case of threatened species. Landscape composition and structure may facilitate or limit individual movement within and among populations. We used a landscape genetics approach to assess the connectivity patterns of the threatened Dupont's lark (*Chersophilus duponti* subsp. *duponti*), considering their genetic patterns and the landscape features associated with its gene flow in Spain. We analysed the genetic relatedness based on 11 species‐specific polymorphic microsatellites on 416 Dupont's lark individuals sampled across peninsular Spain between 2017 and 2019, covering most of the European distribution of the species. To assess the relationship between the landscape composition and the species gene flow, we estimated genetic distance at the individual level (*Dps*). Next, we built a set of environmental surfaces from two time periods (years 1990 and 2018), based on factors such as land use and topography, influencing individuals' movement. We then obtained resistance surfaces from an optimization process on landscape variables. Landscape genetics analyses were done for single and composite surface models for each year separately. Our findings from both time points show that scatter or mosaic‐structured vegetation composed by low agricultural and tree cover and high presence of sclerophyllous shrubs favoured Dupont's lark dispersal, while dense and continuous tree cover, as well as areas of intensive agriculture, were limiting factors. Our results suggest the importance of steppe habitat patches for the species' establishment and dispersal. In addition, our results provide key information to develop conservation measures, including conserving and restoring steppe habitats as scattered and/or mosaic‐structured vegetation that could warrant the connectivity and persistence of Dupont's lark populations.

## Graphical abstract

Landscape genetics analyses have shown for the first time in Dupont's lark, that landscape areas with scatter/mosaic structured vegetation composed by high presence of sclerophyllous shrubs and low agricultural and/or tree cover favour Dupont's lark dispersal, while dense and continuous tree and/or agricultural cover, limit it.

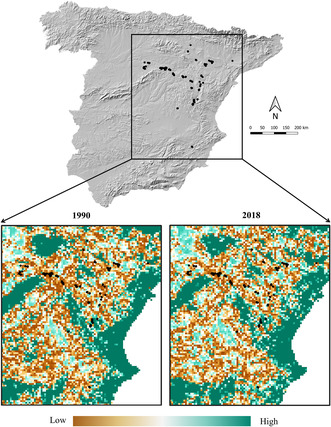

## 1 INTRODUCTION

From an ecological perspective, dispersal influences the distribution and abundance of species and the dynamics and persistence of populations. Besides, from an evolutionary perspective, dispersal affects processes like local adaptation and speciation since it determines the patterns of gene flow among populations (Dieckman et al., 1999; Peninston et al., 2023). Habitat quality, composition and structure are important factors affecting the dispersal, establishment and survival of individuals and hence, populations (Sunny et al., 2022). Individual dispersal, genetic diversity and gene flow, together with effective reproduction, are crucial for the viability of species (Balkenhol et al., 2015; Wittische et al., 2019) and their potential to adapt to environmental changes (Blair et al., 2013; Connor et al., 2022). The environmental features and habitat heterogeneity that define a landscape greatly influence dispersal patterns in most species, because they either facilitate or restrict the movement of individuals (Holderegger & Wagner, 2006). In turn, landscape connectivity and individual dispersal ability are factors that influence population differentiation, yielding the two most common patterns. One, isolation by distance (IBD; Wright, 1943), entails increasing differentiation among populations as geographic distance increases; and the other, isolation by resistance (IBR; McRae, 2006), where population differentiation correlates with environmental resistance to individual movement while considering all possible pathways connecting them (McRae & Beier, 2007). Habitat fragmentation and loss modify the structure and composition of the landscape, creating habitat patches of different sizes, degrees of connectivity and spatial variability of resource availability (e.g. food, shelter). Some likely ecological and evolutionary consequences of habitat fragmentation and loss are declines in population size and genetic diversity and higher genetic structure (Alcaide et al., 2009; Balkenhol et al., 2015; García et al., 2021; Latorre‐Cardenas et al., 2021), leading to local extinctions (Caizergues et al., 2003), which are expected to be more severe in species with restricted dispersal and strict habitat requirements.

Genetic methods are commonly used to indirectly estimate migration rates and effective dispersal, which have the advantage of capturing information about the movement of genes (Fukuda et al., 2022; Koopman et al., 2007). Specifically, the field of landscape genetics focuses on evaluating the influence of landscape features (composition, structure and matrix quality) on microevolutionary processes (i.e. gene flow, genetic drift and selection), at the individual or population level (Balkenhol et al., 2015; Manel et al., 2003; Storfer et al., 2007). Landscape genetics combines landscape ecology, population genetics and spatial statistics to determine the effects of geographic distance and landscape attributes on genetic diversity, structure and gene flow (Baden et al., 2019; Flores‐Manzanero et al., 2019; Latorre‐Cardenas et al., 2021; Shirk et al., 2017; Storfer et al., 2007). It allows the evaluation of functional connectivity, that is, the association between the genetic distance of genetically differentiated populations and the landscape characteristics. To achieve this, resistance surfaces are frequently used, and based on a variety of landscape features like vegetation, topography and climatic and anthropogenic variables, among others, to evaluate their relationship with genetic distances (IBR; McRae, 2006). The use of resistance models not only provides a means to assess the impact of the environment on population genetics but also provides insights into landscape management (e.g. design of corridors, detection of barriers), with applications to deal with current and future landscape changes and the maintenance and conservation of species (Balkenhol et al., 2015; Borokini et al., 2021; Shirk et al., 2017).

Dupont's lark (*Chersophilus duponti* subsp. *duponti*) is a passerine species whose distribution is restricted to Spain and north of Africa (Morocco, Algeria and Tunisia; Suárez, 2010). It is classified as ‘Vulnerable’ globally (BirdLife International, 2022) and as ‘Endangered’ in Spain (BOE Orden TED/339/2023). Furthermore, the species shows a markedly restricted and fragmented distribution, with a metapopulation structure (García‐Antón et al., 2021; Vögeli et al., 2010). It is considered a sedentary and extremely habitat‐selective species, with marked preference for flat (<15% slope) natural shrub steppes (García‐Antón et al., 2019, 2021; Gómez‐Catasús et al., 2016). Such habitat selectivity makes it very sensitive to habitat changes, and thus, a bioindicator of habitat quality (Reverter et al., 2023). Iberian shrub‐steppes are flat treeless areas in dry and semi‐arid environments dominated by small shrubs (<40 cm), like *Genista pumila*, *G. scorpius*, *Thymus* spp., *Lavandula latifolia* and *Satureja intricata* (Zurdo et al., 2021), and with forbs and grasses (dwarf shrub steppes) (Ollero & van Staalduinen, 2012). These habitats are currently experiencing strong degradation (Morales & Traba, 2013; Werger & van Staalduinen, 2012; Zurdo et al., 2021), mainly due to landscape transformation caused by human activity (e.g. agriculture intensification, wind farms and extensive grazing abandonment; Gómez‐Catasús et al., 2021; Reverter et al., 2021). The result of these activities is habitat fragmentation and loss, with an increasing number of small steppe patches, which significantly limits the availability of Dupont's lark suitable habitat. Indeed, such habitat deterioration is one of the main factors of its Iberian range contraction (ca. 36% in the last two decades) and population decline (ca. 2.3% per year; Reverter et al., 2023). Recent estimations consider less than 2300 males in the whole Spanish population, with an effective population size of around 600–1300 pairs (Reverter et al., 2023). Although the impact of habitat degradation on genetic diversity is less evident in birds in comparison with other vertebrate species because of their higher dispersal abilities (Alcaide et al., 2009; Burgess & Garrick, 2020; Canales‐Delgadillo et al., 2012; Coster et al., 2019; Ferrer et al., 2016; Mulvaney et al., 2021), bird species with restricted dispersal and strict habitat requirements are prone to be genetically affected by habitat fragmentation and loss, often exhibiting significant population structure and low genetic variation (Caizergues et al., 2003; Jensen et al., 2019; Khimoun et al., 2017; Klinga et al., 2019).

Previous studies reported variable levels of genetic structure across the range of Dupont's lark, depending on the geographic scale of analysis and the informativeness of the markers used. Mitochondrial analyses at a range‐wide scale found that the Spanish clade encompasses six closely related haplotypes, most of them shared across the Iberian Peninsula (García et al., 2008); thus, no meaningful patterns of mitochondrial differentiation were detected. Microsatellite analyses reported moderate but statistically significant genetic differentiation for the Spanish populations (Méndez, Tella, & Godoy, 2011), rejecting the hypothesis of one single panmictic population. Some of the genetic clusters identified were unique for certain Spanish regions while others were shared among them with high gene flow. Such genetic structure partially coincides with five ecogeographic regions that differ in topography, climate, landscape distribution and amount of suitable habitat within and among clusters (Laiolo & Tella, 2006a, 2006b). The fact that the Dupont's lark populations show both certain structure in genetic clusters and shared mitochondrial haplotypes among regions indicates the existence of historical gene flow, while coalescent‐based models and departures from mutation‐drift equilibrium support a recent reduction in gene flow attributable, at least in part, to the transformation of steppes in the last decades (Méndez, Tella, & Godoy, 2011). This was recently corroborated by a study monitoring temporal trends in population genetic diversity and structure over the last 5–7 generations (Bustillo‐de la Rosa et al., 2022). Authors found signs of genetic erosion and temporal changes in gene flow, which was overall high although variable among regions, and highly dependent on landscape‐mediated connectivity among populations. In fact, the landscape configuration can also act as a potential barrier in this species, as shown by a study of song dissimilarities (Laiolo & Tella, 2006b), whereas suitable habitat patches work as stepping‐stones connecting inhabited areas, i.e. habitat patches identified as suitable for the species establishment and movement among occupied patches at distances up to 20 km (García‐Antón et al., 2021).

Despite the importance of landscape on fragmentation and isolation among populations shown by the aforementioned studies, its relationship with genetic connectivity and gene flow has not been explicitly assessed. Furthermore, the demographic and genetic negative effects associated with population isolation (e.g. population size decline, genetic bottlenecks, diversity loss, genetic drift) can have crucial ecological and evolutionary consequences on, among others, metapopulation dynamics and local adaptation, increasing extinction risk especially in low dispersal specialist species (Peninston et al., 2023; Wong et al., 2023). Indeed, isolated populations resulting from land‐use changes are of major concern in evolutionary biology, conservation and human and animal health (Vázquez‐Domínguez et al., 2024). Therefore, and considering the current habitat modification of natural steppes and the reported decline of the species' populations, it is urgent to evaluate Dupont's lark functional connectivity and determine the landscape features associated with its dispersal and survival.

The aim of this study was to assess the relationship between the landscape composition and structure and the species' gene flow, identifying the main environmental factors influencing connectivity patterns of this threatened species. To this end, we considered two time periods to account for the potential time lag between landscape modifications and its genetic consequences over time. Based on Dupont's lark habitat preferences and restricted dispersal (Gómez‐Catasús et al., 2016; Suárez, 2010), we predicted that connectivity would be facilitated by the presence of steppe patches, characterized mainly by pasture and small shrubs. In contrast, that habitat where vegetation is conspicuously absent, with dense forests, or with agricultural fields would restrict movements. Regarding the anthropogenic landscape modification, potential differences at the two times evaluated in the landscape variables identified as influencing gene flow could be expected. Our results can provide key information to aid in slowing the current process of habitat degradation, further isolation and potential extinction of the species. Namely, for addressing new insights into conservation measures, including the identification of habitat patches as suitable environmental corridors among populations that could conserve and improve the genetic diversity, connectivity and persistence of Dupont's lark populations. Considering the Dupont's lark as a bioindicator of steppe areas, identifying the key landscape variables favouring gene flow and survival can provide general information regarding ecological management of related steppe species.

## 2 MATERIALS AND METHODS

### 2.1 Study system and sampling

**FIGURE 1:**
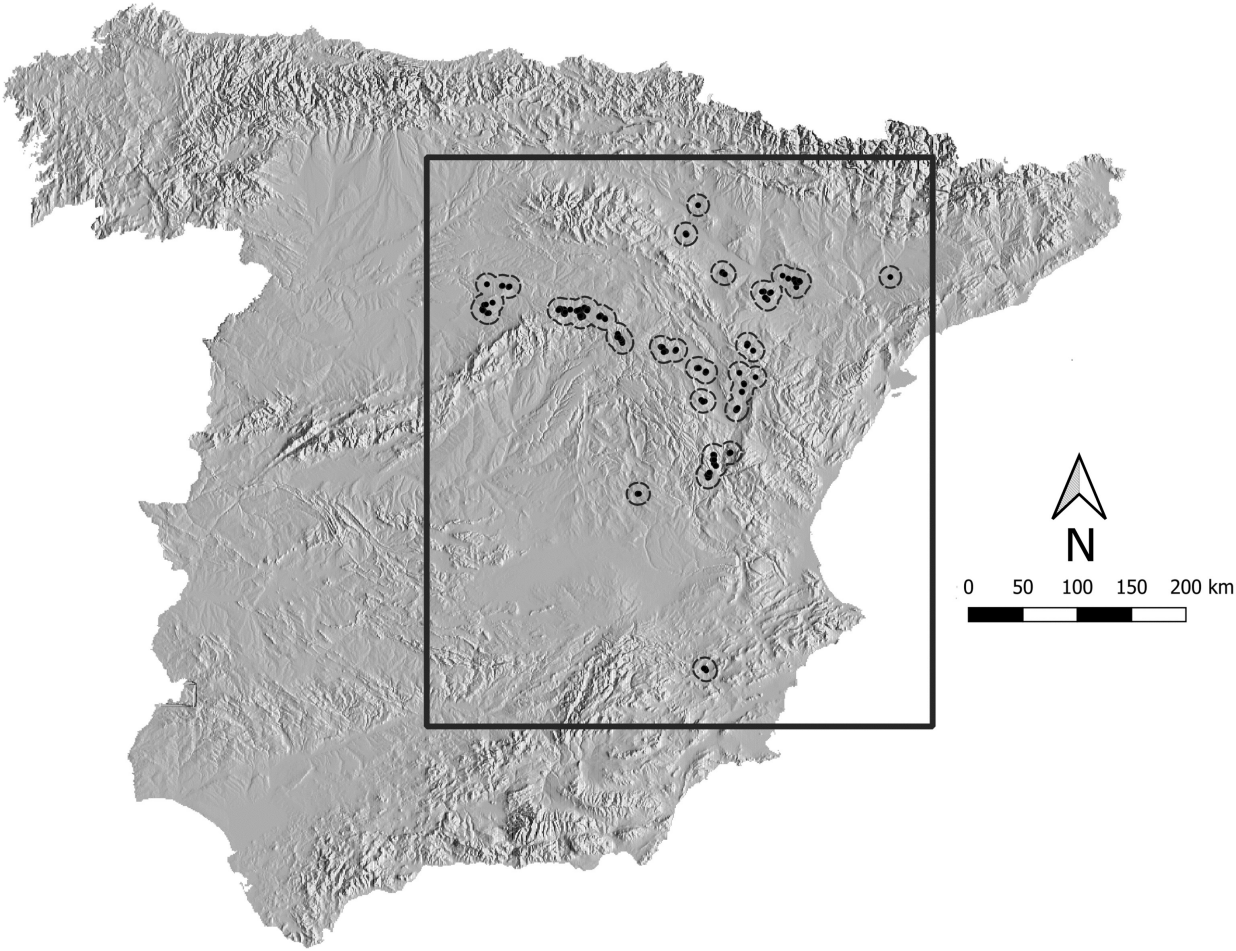
Map showing the sampling localities of Dupont's lark males (black dots) in Spain (*N* = 416) during the breeding season (2017–2019). The dashed line depicts a 10 km buffer around each sample and the square polygon comprises the entire study area.

**FIGURE A1:**
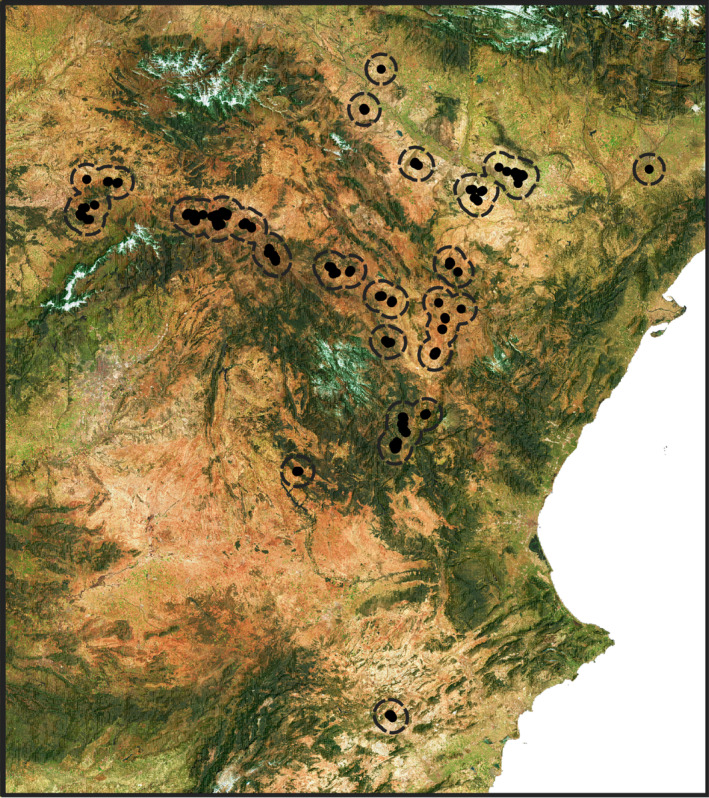
Map showing the sampling localities of Dupont's lark males (black dots) in Spain (*N* = 416) during the breeding season (2017–2019) within the study area. The dashed line depicts a 10 km buffer around each sample. Green areas represent vegetated areas; brown ones comprise unvegetated and crop areas.

Dupont's lark individuals were sampled during the breeding season across peninsular Spain between 2017 and 2019 (Figure 1, see Figure A1 in Appendix A for the main landscape features within the study area), considering the locations where its presence was previously registered during the II National Census of the species (2004–2007; Suárez, 2010). Captures were carried out using individual spring‐traps baited with mealworms (*Tenebrio molitor*), and fieldwork was performed exclusively by a team specialized in Dupont's lark. We also used Dupont's lark recordings played through speakers to attract individuals; thus, sampling was biased towards adult males as described by Laiolo et al. (2007). Every individual captured was metal‐ringed for identification and to avoid resampling. UTM coordinates of every individual captured were recorded using a GPS. We collected blood samples from the jugular or brachial vein using 0.5 mL insulin syringes and capillary tubes; samples were stored in pure ethanol and transported to the laboratory for genetic analyses. All individuals were released at the point of capture, keeping handling time as short as possible to avoid potential stress. The protocol for capture, handling, blood‐sampling and release was approved by the Ethical Committee for Animal Experiments of the Universidad Autónoma de Madrid (CEI80‐1468‐A229).

### 2.2 Microsatellites amplification and genetic analyses

In the present study, we used the microsatellite loci genotypes obtained by Bustillo‐de la Rosa et al. (2022) where the DNA extraction protocol and PCR conditions are explained. Briefly, DNA was extracted from blood samples which were genotyped at 12 polymorphic microsatellite loci isolated in *C. duponti* (Méndez, Prats, et al., 2011). One locus showed no polymorphism and was removed from further analyses (Bustillo‐de la Rosa et al., 2022). Amplifications were performed in one multiplex panel using four dyes (FAM, PET, NED, VIC) in a 10 μL total volume containing: 25–50 ng of template DNA, 1× QIAGEN Multiplex PCR Master Mix and 0.1 μM of each forward and reverse primers. PCR products were processed using capillary electrophoresis with an ABI 3730 by the Unidad de Genómica, Universidad Complutense de Madrid, Spain (https://www.ucm.es/gyp/genomica). Electropherograms were scored using Geneious v.10.2.3 (https://www.geneious.com). Negative controls (i.e. ddH_2_O) were included in all runs and 7% of the samples were re‐amplified and re‐scored to confirm our genotypes and to estimate the genotyping error rate, which was less than 1% (see Table B1 in Appendix B for further information about the genotyping error rate per locus). The original data set was evaluated with Genepop on the web (Raymond & Rousset, 1995) and results showed that no microsatellite loci exhibited evidence of significant deviation from Hardy–Weinberg or linkage disequilibrium.

To avoid a potential bias from non‐random sampling and overrepresentation of allele frequencies in our data due to the presence of closely related individuals (Waples & Anderson, 2017), we assessed relatedness between pairs of individuals with ML‐Relate (Kalinowski et al., 2006). Less than 1% of pairs exhibited a first‐order relationship (full‐sibling, parent‐offspring), thus all individuals were kept for analyses. Genetic population structure of Dupont's lark has been previously addressed with different genetic markers (Bustillo‐de la Rosa et al., 2022; García et al., 2008; Méndez, Tella, & Godoy, 2011), showing that there are both unique and shared clusters in Spain with some degree of gene flow among them. Thus, landscape genetic analyses were performed at the individual level, calculating genetic distance between all pairs of individuals, increasing the likelihood to find genetic differences at finer scales and to detect novel barriers to gene flow (Mulvaney et al., 2021; Pavlacky Jr. et al., 2009). We estimated the proportion of shared alleles (*Ps*) with the function *propShared* in *adegenet* and subtracted those values from one to obtain a genetic distance measure (*Dps* = 1 − *Ps*), which is an adequate metric for performing individual‐based genetic distance estimates and useful for fine‐scale landscape genetic analyses (Flores‐Manzanero et al., 2019; Kimmig et al., 2020; McCluskey et al., 2022; Shirk et al., 2017). We tested for IBD with a Mantel test between genetic (*Dps*) and geographic distances; geographic (Euclidean) distance was estimated with GenAlEx v.6.5 (Peakall & Smouse, 2006, 2012). Significance was obtained using 1000 permutations.

To incorporate explicit information about the species dispersal distances and address potential movement thresholds, we also performed Mantel correlograms based on mean dispersal distances (Storfer et al., 2007). We selected distance class boundaries of 5 and 20 km described for Dupont's lark resident movements and juvenile dispersal, respectively, which reflect sensible biological classes (García‐Antón et al., 2021). We employed an individual‐based method using multi‐locus spatial autocorrelation analyses in GenAlEx 6.5 (Banks & Peakall, 2012; Peakall & Smouse, 2006) to test the absence of spatial genetic structure. This method provides a measure of genetic correlation as a function of geographic distance. The method requires as inputs a geographic and a genetic distance matrix and provides an estimate of the autocorrelation coefficient (*r*) for each distance class. The null hypothesis (no spatial structure; *r* = 0) was obtained by 1000 random permutations of *r* values (random shuffling of all individuals among the geographic locations). If the calculated *r* values fall outside the 95% confidence interval bounding the null hypothesis, significant spatial genetic structure is inferred. We also defined the 95% confidence interval for each estimate of *r* by 999 bootstraps within each distance class (Peakall et al., 2003).

### 2.3 Landscape data

To evaluate the influence of landscape features on gene flow, we considered environmental predictors that could influence Dupont's lark movement based on current knowledge of the species' biology and habitat preferences (Gómez‐Catasús et al., 2016; Méndez et al., 2014; Suárez, 2010), and which were previously used to model the potential presence of the species (Table 1; García‐Antón et al., 2019) and movements (García‐Antón et al., 2021). Anderson et al. (2010) suggested that the grain size (i.e. pixel size) of landscape data should be based on the distance of resident movements described for the study species. An estimate of 5 km has been recognized as the distance threshold for resident movements in Dupont's lark and for subpopulation boundaries (García‐Antón et al., 2021), as well as the limit of cultural similarity in song structure among individuals (Laiolo, 2008) and from capture–recapture data (Pérez‐Granados & López‐Iborra, 2015). Hence, we used a grain size of 5 km.

**TABLE 1:** Land use and topographic variables (% cover and mean values in a 5 × 5 km cell respectively) considered for the landscape analyses.

Variable	Description	Expected effect on gene flow
Land use		
Urban^a^	Artificial surfaces (111,112,121,122,123,124,131,132,133,141,142)	Not favourable
Agr_dry	Dryland crops (211)	Not favourable
Agr_nat	Crops and areas of natural and seminatural vegetation (243)	Not favourable
Agr_tree	Farming and forest (241, 242, 244)	Not favourable
Pasture	Natural grassland (321)	Favourable
Shrub_scle	Sclerophyllous shrub (323)	Favourable
Shrub_other	Other types of shrubs (322, 324)	Not favourable
Forest	Forest areas (311, 312, 313)	Not favourable
Topography		
Elevation	Metres above sea level	Favourable at low elevation
Slope	Inclination angle	Favourable at low slopes

*Note*: Land use variables were obtained from CORINE 2018 and 1990 (codes between brackets refer to the surfaces considered for each classification); except for elevation and slope that were from DTM raster 25 m. The predicted effect of each landscape variable in terms of limiting (not favourable) or facilitating (favourable) dispersal (see Methods in main text) is indicated.

^a^ The urban surface was used only for the 2018 models.

To build the environmental surfaces, we created a 5 × 5 km grid cell across Peninsular Spain and estimated the percentage cover of each landscape variable per 5 km pixel. Next, to avoid potential bias due to an overextended study area, we first created a buffer of 10 km around each sampling location (García‐Antón et al., 2021). Then, we defined the study area as the surface surrounding all buffer locations. Last, we clipped all the surfaces estimated across the Peninsular Spain using the study area as a mask (Figure 1) and keeping the same grid cells resolution.

Contemporary land use layers were obtained from CORINE data available for the year 2018 (https://land.copernicus.eu/). However, the effect of landscape features on genetic processes cannot always be detected due to the time lag between environmental changes and the species' genetic response (Epps & Keyghobadi, 2015; Pavlacky Jr. et al., 2009). Moreover, regarding anthropogenic landscape changes, the analysis of temporal effects is hampered by the fact that the alteration is often a continuous process over a considerable period, making it difficult to determine a precise date for such alterations. Since theory predicts that negative genetic effects would increase over time with increasing numbers of generations (Young & Clarke, 2000), we also evaluated the effect of landscape features on gene flow using the oldest available landscape data set (CORINE land cover from 1990). Accordingly, it will allow us to assess whether the effect of landscape variables on gene flow is persistent over time (i.e. landscape variables affecting gene flow in both time points). For this, we reclassified the CORINE original 1990 and 2018 land cover categories into 12 different surface, selecting the variables we considered could limit or facilitate Dupont's lark dispersal and that were adequate to define both suitable breeding areas (as in García‐Antón et al., 2019) and potential corridors (Table 1): urban, dryland farming (Agr_dry), other farming (Agr_other), farming+natural mosaic (Agr_nat), farming+forest mosaic (Agr_tree), natural grassland (Pasture), sclerophyllous shrubs (Shrub_scle), other shrubs (Shrub_other), forest, scarce vegetation, unvegetated and water bodies (Table 1). Sclerophyllous shrubs were differentiated from other shrubs as they have specifically been previously described as favourable for the species presence. Furthermore, ‘Shrub_other’ comprises moors and heathlands (Code 322 of CORINE) and transitional woodland/shrub (Code 324), which represent closed vegetation of great height, defined as unfavourable for the species. In contrast, sclerophyllous shrubs included bushy sclerophyllous vegetation mixed with some bare ground (Code 323 of CORINE), considered a priory as potentially favourable for Dupont's lark (Table 1).

**FIGURE 2:**
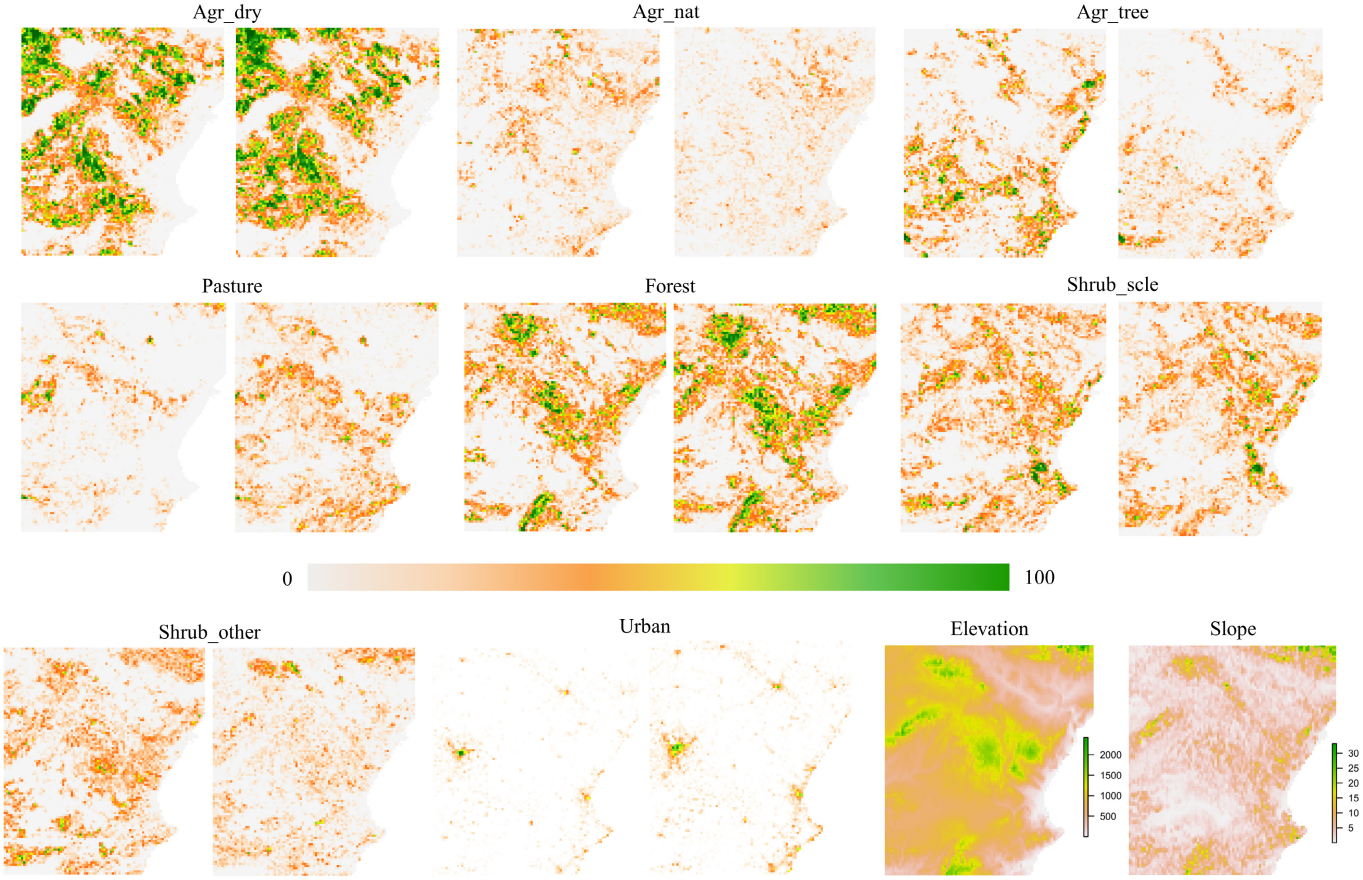
Raster layers (5 × 5 km) showing the different land uses considered for the resistance models for the Dupont's lark in Spain, based on the CORINE data 1990 and 2018 (left and right respectively). The colour bar shows the percentage cover of each land use, the elevation (in metres) and the slope (in percentage) for the study area.

We used vector layers to calculate cover (expressed as percentage) of every surface at each grid cell, based on which we built raster layers for every land use. For the topographical variables, we obtained data for elevation, its coefficient of variation and slope from the National Centre of Geographic Information with a 25 m resolution (DTM 25 m; https://www.cnig.es/). Topographic values were incorporated to the 5 × 5 km grid with the bilinear interpolation method. All raster cells that fell outside the Spanish geographic border were kept as ‘NoData’ values and were not considered during the optimization process and analyses. Next, variables that were mostly absent across the study area, namely those that had values of 0 in 80% or more of the pixels, were removed (scarce vegetation, unvegetated, water bodies and the slope's coefficient of variation for both time points, whereas urban was removed only for the 1990 data). To avoid collinearity, we calculated the variance inflation among all variables using the *VIF* function in R. Those variables with high collinearity were individually removed until none showed VIF > 5 (Fox & Monette, 1992). Only ‘other farming’ for both 1990 and 2018 was eliminated, resulting in a final data set with 10 variables for 2018 and nine for 1990 (Figure 2; Table 1).

### 2.4 Landscape genetic analyses

We performed landscape genetic analyses for both data sets (1990 and 2018) separately. We applied the optimization framework developed by Peterman et al. (2014) to determine the resistance values of our environmental surfaces with no a priori assumptions about the scale and direction of the resistance relationship. The resistance surfaces optimization framework (Peterman, 2018; Peterman et al., 2014) uses Ricker and Monomolecular equations to transform data, in combination with linear mixed‐effects models and genetic algorithms, not making a priori assumptions regarding the scale and direction of the resistance relationship. The Monomolecular and Ricker are two exponential‐based functions used for ecological modelling, differing in the curve shape of the relationship they are modelling. This curve is mainly determined by shape and magnitude parameters, which produce a saturating exponential (growth or decay) curve for the Monomolecular function, and a hump‐shaped curve (skewed to right or left) for the Ricker function (Bolker, 2008). During the optimization process, the genetic algorithm searches all possible combinations of these parameters for transforming resistance surfaces, denoted by ‘r’ (Peterman, 2018; Peterman et al., 2014). We determined landscape resistance values that best explained pairwise genetic dissimilarity (genetic distance) between individuals with ResistanceGA (Peterman, 2018), testing both IBD and IBR scenarios (Peterman, 2014, 2018). ResistanceGA explores the parameter space and maximizes the relationship between pairwise landscape distances (least‐cost or resistance) and the genetic distance (in this case *Dps*), using a genetic optimization algorithm (GA, Scrucca, 2013). It also uses generalized linear mixed‐effect models fitted using maximum likelihood population effects parameterization (MLPE) to account for the non‐independence error associated with pairwise distances (Clarke et al., 2002).

The optimization process comprises several steps. First, continuous landscape variables were individually transformed based on Ricker and Monomolecular functions (eight transformations in total per variable; Bolker, 2008) using the *GA.prep* function with the default parameters. Transformations are performed to maximize the relationship between pairwise landscape and genetic distances. Next, surfaces were individually optimized estimating pairwise effective distances among individuals using the *commuteDistance* function with *gdistance* in R (van Etten, 2014), which evaluates the length of a random roundtrip move that connects two nodes; it uses an eight‐neighbour connection scheme to assess connectivity (Flores‐Manzanero et al., 2019; van Etten, 2014). We used Akaike's information criterion value (AIC) during the optimization process as the objective function, which was determined from linear mixed‐effects models (lmem) fitted by the MLPE parameterization using lme4 in R (Bates et al., 2014).

We used *Dps* as the response variable, the pairwise resistance distances for each landscape surface as the dependent variable, and the identity of pairwise combination of individuals sampling localities as random effect. The best‐optimized resistance surfaces were estimated based on the AIC corrected for small/finite populations (AICc) value (Burnham & Anderson, 2002). By default, ResistanceGA also calculates a distance‐only (IBD) and a null model to test model performance. To control for the potential bias from the sampling design (type I error), and to test the robustness of our models, we conducted a pseudo bootstrap analysis (*Resist.boot* function in R). We randomly selected 75% of the samples without replacement and optimized surfaces were then fit to the subsample data. Using 10,000 iterations, we calculated for each model the average rank, average model weight, average marginal *R* ^2^ (proportion of the variance explained by the fixed factors) and the proportion (percentage) in which a surface was chosen as the best model. The resulting surfaces were then chosen to build composite surfaces and run a multivariable optimization. Optimization (single and composite surface models) was conducted twice to ensure convergence (Peterman, 2018). Finally, the best replicates (overall best AICc) of single and composite models were selected to run a bootstrap model selection to obtain the average model rank, average model weight and the top ranked model (being the models with highest percentage the ones identified as best models).

## 3 RESULTS

### 3.1 Population genetics and fine‐scale genetic structure

A total of 416 Dupont's lark males were captured during this study (11% of the estimated number of Dupont's lark males in Spain; Traba et al., 2019; Figure 1), and their DNA successfully amplified for 11 microsatellite loci.

**FIGURE 3:**
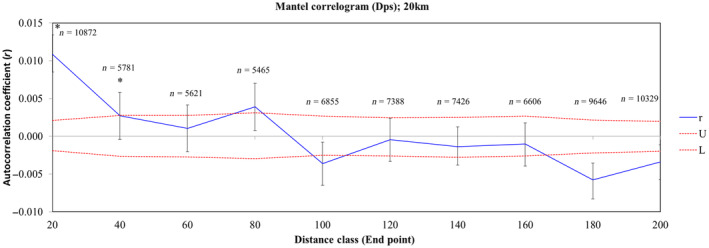
Correlogram showing the genetic correlation *r* (mean value and 95% confidence error bars by bootstrapping) as a function of 20 km distance bins (10 distance classes, 20 km each up to 200 km). Red lines are the 95% CI of a null hypothesis of random distribution of genotypes (U, upper; L, lower), calculated using 999 permutations. Asterisks indicate statistically significant values. Sample sizes (*n*; number of pairs compared) of each distance class are also indicated.

**FIGURE C1:**
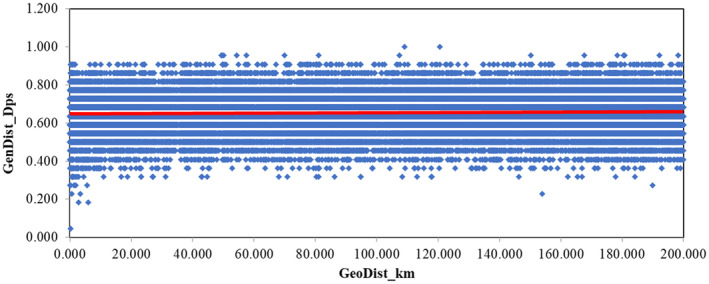
Mantel test graph showing the relation between genetic distance (*Dps*) and geographic distance (km) among Dupont's lark pair of individuals in Spain.

The Mantel test showed a weak positive albeit significant correlation between genetic (*Dps*) and Euclidean geographic distances (*r* = .049; *p* = .001; see Figure C1 in Appendix C for the graph representation of the Mantel test). On the other hand, Mantel correlograms showed positive and significant correlations (*r* = .004–.019; *p* < .05) up to a distance of about 20–30 km between samples, where individuals tended to be genetically more similar than expected by chance (Figure 3). As the 5 and 20 km correlograms showed similar patterns, only the 20 km one is shown.

### 3.2 Landscape genetics

**FIGURE 4:**
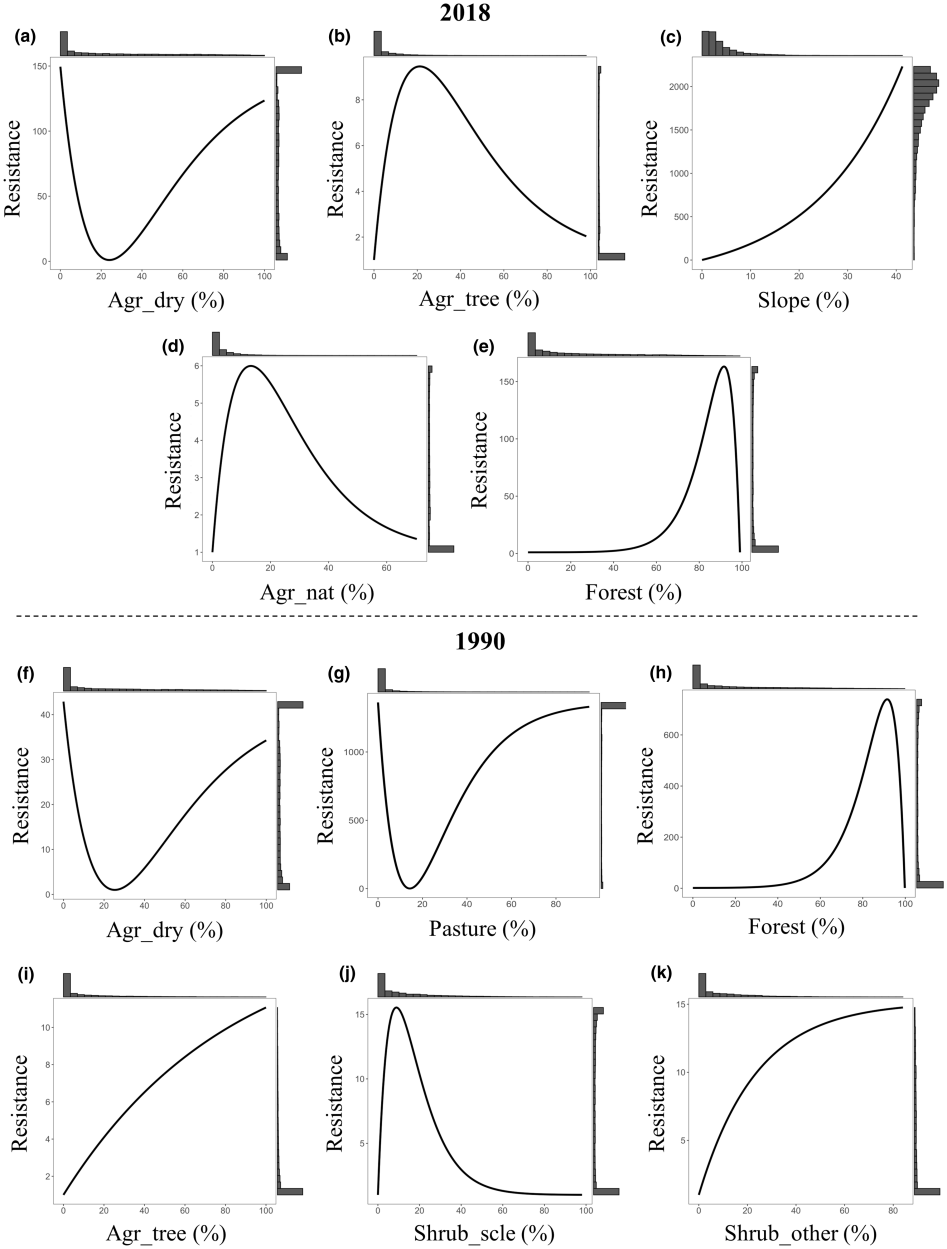
Single surface optimization response curves obtained with ResistanceGA for the relationship between landscape features and genetic distance (*Dps*) for Dupont's lark in Spain in 2018 (panel above) and 1990 (below). Each curve represents the resistance cost imposed by each landscape variable after the optimization procedure. Histograms represent the frequency of each resistance value. The transformation equation and the model support for each variable are described in Table 2.

Optimization and bootstrap model selection for the single and composite surfaces from ResistanceGA analyses showed that landscape variables that performed better than geographic distance alone (i.e. IBD) differed between 1990 and 2018. Results for 2018 data set showed that the two best supported models (increment in AIC less than two among them) after the bootstrapping were dry farming (Agr_dry) and farming and forest mosaic surface (Agr_tree) (39.98% and 29.74% of the times respectively; Table 2). These two variables were defined by Inverse‐Ricker and Ricker functions respectively. The following well‐supported model was Composite 5 (7.17%; ΔAIC = 2.5) which included the two variables: Agr_dry and Agr_tree, with a higher contribution of the former (68%) than the latter (32%) (Table 2, see Tables D1 and D2 in Appendix D for parameter estimates from mixed effects models). Three other models had moderate support: Slope (6.83%), Agr_nat (6.32%) and Forest (5.55%) (3.2 < ΔAIC < 4.9), while the remaining models, comprising also the geographic distance (ΔAIC = 7.4) showed low levels of support (<2%; ΔAIC 5.7–28.1). Resistance results indicated that moderate extent (10%–40%) of dry arable land (Agr_dry) had the lowest level of resistance for Dupont's lark, with increasing resistance below and above these values (Figure 4a). Conversely, the agriculture‐forest mosaic (Agr_tree) showed that moderate levels (10%–40%) had the highest resistance (Figure 4b). Moreover, these two variables were significant predictors of genetic distance in the generalized linear mixed‐effects models (see Tables D1 and D2 in Appendix D). Flat areas showed low resistance, with resistance increasing exponentially as the slope increased (Figure 4c); also, resistance peaked around 10%–20% Agr_nat cover (Figure 4d). Regarding Forest, areas with less than 60% forest cover showed little or no resistance, while areas with >80% were classified with the highest resistance (Figure 4e). All other single and composite resistance surfaces showed low or null contribution to gene flow (Table 2).

**TABLE 2:** Bootstrap model selection results for the single and composite surfaces that resulted from multisurface optimization on genetic distance (*D*_PS_) for Dupont's lark in Spain.

Surface	Equation	*k*	Average AICc	ΔAICc	Average weight	Average rank	*R* ^2^m	*R* ^2^c	Top ranked %
2018									
Agr_dry	Inverse Ricker	4	−107,816	0	3.20E−01	3.1495	0.0068	0.1409	39.98
Agr_tree	Ricker	4	−107,815	1	2.39E−01	3.5543	0.0067	0.1395	29.74
Composite_5	NA	7	−107,813.5	2.5	1.03E−01	4.6898	0.0085	0.1422	7.17
Slope	Inverse—Reverse Monomolecular	4	−107,812.8	3.2	8.80E−02	4.809	0.0040	0.1366	6.83
Agr_nat	Ricker	4	−107,812.2	3.8	7.36E−02	5.1604	0.0049	0.1380	6.32
Forest	Reverse Ricker	4	−107,811.1	4.9	6.70E−02	6.2566	0.0035	0.1367	5.55
Urban	Ricker	4	−107,810.3	5.7	3.99E−02	6.6989	0.0038	0.1369	1.77
Geographic distance	NA	2	−107,808.6	7.4	2.38E−02	7.7399	0.0022	0.1356	1.18
Shrub_scle	Inverse Ricker	4	−107,806.8	9.2	1.66E−02	9.7265	0.0036	0.1375	1.15
Composite_4	NA	10	−107,807.9	8.1	1.38E−02	8.5797	0.0086	0.1417	0.29
Shrub_other	Reverse Ricker	4	−107,806.2	9.8	5.64E−03	10.5212	0.0025	0.1360	0.02
Pasture	Monomolecular	4	−107,805.9	10.1	4.32E−03	10.9544	0.0029	0.1362	0
Elevation	Inverse—Reverse Monomolecular	4	−107,805.6	10.4	4.65E−03	11.2896	0.0021	0.1355	0
Composite_3	NA	13	−107,800.7	15.3	2.06E−04	12.3773	0.0089	0.1423	0
Composite_2	NA	16	−107,795.2	20.8	8.15E−06	14.5888	0.0074	0.1405	0
Composite_1	NA	19	−107,787.9	28.1	2.94E−07	15.9041	0.0079	0.1412	0
1990									
Agr_dry	Inverse Ricker	4	−107,822	0	4.04E−01	2.1860	0.007	0.1405	50.88
Pasture	Inverse Ricker	4	−107,817.6	4.4	1.52E−01	4.8373	0.008	0.1415	15.99
Forest	Reverse Ricker	4	−107,817.3	4.7	1.45E−01	5.2251	0.004	0.1373	15.62
Agr_tree	Monomolecular	4	−107,814.6	7.4	5.97E−02	6.7395	0.005	0.1381	5.51
Shrub_scle	Ricker	4	−107,813.8	8.2	4.66E−02	7.1594	0.004	0.1368	4.18
Shrub_other	Monomolecular	4	−107,815.8	6.2	5.79E−02	6.1606	0.004	0.1381	3.86
Slope	Inverse‐Reverse Monomolecular	4	−107,816.5	5.5	4.42E−02	5.3206	0.004	0.1372	2.24
Geographic distance	NA	2	−107,812.5	9.5	2.10E−02	8.1263	0.002	0.1357	1.17
Composite 6	NA	7	−107,818.3	3.7	5.59E−02	4.1506	0.006	0.1394	0.46
Agr_nat	Reverse Ricker	4	−107,810.4	11.6	6.76E−03	10.0167	0.004	0.1380	0.09
Composite 5	NA	10	−107,811.7	10.3	2.04E−03	8.7421	0.007	0.1396	0
Elevation	Inverse‐Reverse Monomolecular	4	−107,810.3	11.7	4.97E−03	10.3590	0.002	0.1356	0
Composite 4	NA	13	−107,805.3	16.7	8.95E−05	12.0724	0.007	0.1395	0
Composite 3	NA	16	−107,797.2	24.8	1.51E−06	13.9167	0.006	0.1393	0
Composite 2	NA	19	−107,790	32	5.73E−08	14.9883	0.007	0.1406	0
Composite 1	NA	22	−107,783.7	38.3	1.85E−09	15.9994	0.007	0.1402	0

*Note*: Landscape variables evaluated from 2018 and 1990. The fitted transformation for individual surfaces optimization is also shown. Best‐supported models are indicated by higher ‘top ranked %’ (number of times the model chosen performed better than the rest considering bootstrapping). *k* = number of parameters fit in each model plus the intercept. Average weight = (the probability that a model is the best in the model set, averaged over 1000 bootstrap replicates). AICc = Akaike information criterion value generated from the MLPE mixed effects model corrected for small sample size; ΔAICc is the AICc value adjusted for the number of populations sampled and the number of parameters optimized. weight = weight of support for surface *i*, given the surfaces assessed. R^2^m and R^2^c are the marginal and conditional values, respectively, of the fitted MLPE model. Composite surfaces include variables as explained in Table D1 of the Appendix D.

When analysing data from 1990, the best supported model was dry farming (Agr_dry; 50.88%), while two other moderately well‐supported models were Pasture (ΔAIC = 4.4) and Forest (ΔAIC = 4.7) (15.99% and 15.62% respectively; Table 2). Dry farming and Pasture were defined by an Inverse Ricker function, and Forest by a Reverse Ricker function. As in 2018, other three models had moderate support: Agr_tree (5.51%), Shrub_scle (4.18%) and Shrub_other (3.86%). The remaining models, comprising also geographic distance (ΔAIC = 9.5), showed low level of support (<2.24%; ΔAIC 7.4–38.3).

The optimized pattern observed for Agr_dry was the same as in 2018, assigning low resistance only to areas around 20%–30% cover (Figure 4f). Additionally, values associated with Pasture showed lowest resistance close to 20% cover, and high resistance at all other cover percentages (Figure 4g). Forest had also the same pattern in 1990 as in 2018 with low resistance below 60% (Figure 4h). As observed for 2018, lowest resistance values were identified where Agr_tree was absent (Figure 4i), but with a steep increase in resistance as farming and forest mosaic surface rises. Finally, lower resistance values were found with both absence and >40% sclerophyllous shrub surface (Shrub_scle) (Figure 4j), whereas the other shrub surface (Shrub_other) exhibited increasing resistance with increasing shrub cover (Figure 4k). Considering the percentage top ranked models, the two time periods showed consistent resistance patterns for Agr_dry and Forest, but with contrasting effect regarding pasture surfaces.

## 4 DISCUSSION

By using a landscape genetics approach, we were able to discern connectivity patterns of Dupont's lark in Spain with fine detail and at the individual level. Our findings demonstrate how the landscape matrix and composition influence Dupont's lark genetic patterns, identifying how steppe patches (i.e. flat areas covered by low shrubs and pasture) are favourable for its effective dispersal. In contrast, the presence of forest areas, high vegetation and/or intensive agriculture seem to limit gene flow.

The spatial pattern observed where individuals tended to be genetically more similar within a distance of approximately 20–30 km than those further apart strongly supports the proposed stepping‐stone model of connectivity for Dupont's lark (García‐Antón et al., 2021; Méndez et al., 2014) instead of more general models based on long dispersal distances (e.g. Coster et al., 2019). This pattern agrees with the genetic structure observed in Spain, where some genetic clusters are not entirely isolated (Méndez, Tella, & Godoy, 2011). Similar patterns have been identified for other bird species mainly because of their high dispersal capacities (Kleinhans & Willows‐Munro, 2019; Koopman et al., 2007; Kunz et al., 2022). To directly evaluate the landscape influence on the stepping‐stone connectivity, future studies should consider a sampling design that specifically accounts for the habitat features of patches potentially serving as stepping stones (Duforet‐Frebourg & Slatkin, 2016).

Landscape composition and structure changed through time, as can be seen in the overall land use differences (surfaces) between the two time points we evaluated (Figure 2). Such variation highlights the importance of considering different temporal data sets, when available, in landscape genetics to assess individual genetic responses in relation to the landscape changes during the time lag considered (Epps & Keyghobadi, 2015; Landguth et al., 2010; Manel & Holderegger, 2013). Some caution is needed when interpreting the influence of land use surfaces on gene flow, as our model results could be influenced by the differences in the CORINE classification methods. CORINE landcover categories of 1990 were generated from a human photo‐interpretation of satellite images and orthophotos at 1:100,000 scale. In contrast, land use categories for 2018 were generated at a 1:25,000 scale with a generalization of SIOSE 2014, which is an Information System of Land Occupation in Spain, included in the Nation Plan for Territorial Observation. The latter provided higher accuracy in terms of land use polygons delimitation for the whole country (García‐Álvarez & Olmedo, 2022; Martínez‐Fernández et al., 2019). Nonetheless, we are confident of the pattern observed given the concordance with Dupont's lark distribution and life history.

**FIGURE E1:**
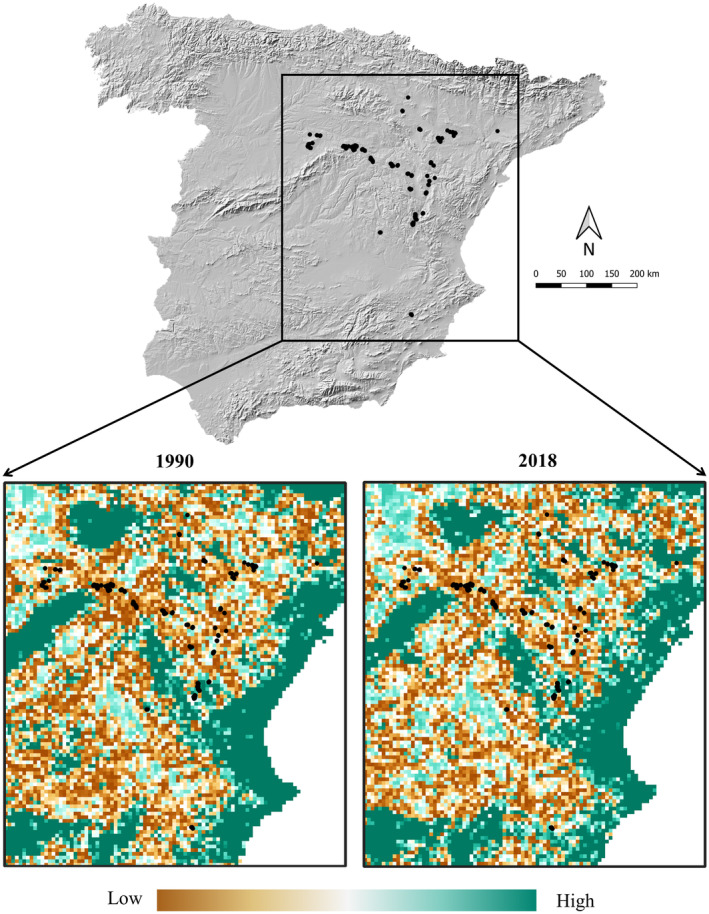
Map (above) showing the sampling localities for Dupont's lark males (black dots) in Spain during the breeding season (2017–2019), represented over an elevation surface. The squared polygon with bold line comprises the study area within Spain. Resistance maps (below) show the optimized resistance surfaces obtained based on the dry farming land use cover in 1990 (left) and 2018 (right). Resistance costs are indicated by the gradient colour bar on the bottom.

Our findings that gene flow in Dupont's lark is best explained by dry farming areas, for the time analysed, notably suggest a persistent pattern over time rather than a temporary effect. Indeed, we show that moderate cover of dry farming facilitates the dispersal of Dupont's lark, acting as a barrier when its cover exceeds certain values, thus potentially acting as a barrier to gene flow in much of the species' range (see Figure E1 in Appendix E for resistance maps). Interestingly, the low resistance values at moderate agriculture cover could be related to fallow lands, which have been described previously as potentially beneficial for Dupont's lark dispersal during the non‐breeding season (Suárez et al., 2006). Moreover, García‐Antón et al. (2019) found that habitat patches where dry farming cover was higher than 40% could be unsuitable habitat for this species.

High resistance costs for forested areas were also identified with both temporal data sets, indicating that gene flow is strongly limited on highly forested areas and by high‐vegetation cover (Agr_tree and Shrub_other). This is consistent with the ecology and habitat preferences of this species (Gómez‐Catasús et al., 2016; Suárez, 2010). Interestingly, the 1990 surfaces were important to identify areas with low pasture cover, namely areas with ≤30% of natural grassland cover mixed with shrub vegetation, together with high cover of sclerophyllous shrubland, as landscape variables facilitating individual movement. This confirms our prediction that steppe patches would be a key habitat supporting gene flow. Therefore, considering the best models obtained for both time periods, landscape features better explaining Dupont's lark dispersal are consistent with those environmental variables described as key factors for its occurrence. Open landscapes with scattered and/or mosaic structured vegetation, including low agricultural surface and dispersed trees combined with high sclerophyllous shrub cover may ease movement. In contrast, areas of intensive agriculture and dense and continuous tree cover function as barriers. This pattern was also showed by Pavlacky Jr. et al. (2009) for the Australian logrunner (*Orthonyx temminckii*), where habitat preferences determining its occurrence were also important for its dispersal.

We acknowledge that our results have potential limitations, first because we only analysed adult males, and this species might exhibit sex and/or age bias in dispersal, whereas association with specific landscape features could differ between sexes (Garza et al., 2005). Secondly, other environmental variables not considered in this study could also influence Dupont's lark gene flow, like habitat quality (i.e. resource availability), patch size, behavioural traits (e.g. idiosyncratic low gene flow due to its sedentary behaviour) or demographic patterns (e.g. lower genetic differentiation and higher genetic diversity for larger population sizes; Kimmig et al., 2020; Méndez et al., 2014). Nonetheless, we were able to identify patterns of landscape heterogeneity influencing Dupont's lark gene flow, and thus, its potential impact on the species dispersal and persistence.

Considering the negative effects of anthropogenic fragmentation of habitats that significantly alter patterns of dispersal and functional connectivity, impacting ecological and evolutionary patterns, diminishing genetic diversity and preventing the spread of adaptive complexes (Peninston et al., 2023; Wong et al., 2023), our findings support the threatened status of Dupont's lark and call for urgent conservation measures. In this study, we have shown for the first time how, despite the potential high dispersal of bird species (suggested by the low relatedness values obtained), Dupont's lark gene flow and future survival are tightly linked to the presence of shrub steppe areas (recognized as a unique and threatened habitat in Europe; Sainz Ollero, 2013), impacted by land use transformations. Previous studies showed that the viability of Dupont's lark overall metapopulation is maintained by the current large size of some populations and the connectivity among them (Bustillo‐de la Rosa et al., 2022). However, the species is suffering a severe decline (Reverter et al., 2023) following a centripetal contraction due to the extinction of peripheral populations. The most peripheral populations are the most affected by habitat fluctuations, given they are smaller and more isolated (García‐Antón et al., 2021; Méndez et al., 2014; Reverter et al., 2023). Moreover, this species is indeed already showing signs of genetic erosion mainly due to habitat loss and fragmentation associated with land use changes (Bustillo‐de la Rosa et al., 2022; García‐Antón & Traba, 2021; Gómez‐Catasús et al., 2018; Méndez, Tella, & Godoy, 2011).

In all, it is imperative to guide management efforts to conserve and restore steppe landscape features, characterized by scattered and mosaic‐structured vegetation with predominance of low sclerophyllous shrublands, which provide both suitable breeding grounds and stepping‐stones for this endangered bird and related steppe species. For instance, by limiting the expansion of intensive crops or afforestation, or recovering extensive livestock grazing, whose decline has a direct impact on steppe habitat because it favours forest recovery following secondary vegetation succession (Martínez‐Valderrama et al., 2021; Reverter et al., 2019; Traba & Pérez Granados, 2022).

## AUTHOR CONTRIBUTIONS

**Daniel Bustillo‐de la Rosa:** Data curation (lead); formal analysis (lead); investigation (equal); methodology (equal); writing – original draft (lead); writing – review and editing (equal). **Adrián Barrero:** Investigation (supporting); writing – review and editing (supporting). **Juan Traba:** Conceptualization (lead); funding acquisition (lead); writing – review and editing (supporting). **Jesús T. García:** Investigation (equal); writing – review and editing (supporting). **Manuel B. Morales:** Writing – review and editing (supporting). **Ella Vázquez‐Domínguez:** Data curation (lead); formal analysis (lead); methodology (equal); supervision (lead); writing – original draft (lead); writing – review and editing (supporting).

## CONFLICT OF INTEREST STATEMENT

The authors have no relevant financial or non‐financial interests to disclose.

## Data Availability

I confirm that the dataset used for this study is available in a Dryad repository: https://datadryad.org/stash/share/rjij39tgv_wT‐bNGUN9xTHGso2T‐nJHrMFmtYthZlCA.

## APPENDIX B

**TABLE B1:** Rate of allele dropout and false alleles for 11 loci on Dupont's lark individuals.

Locus	A112	B107	D115	D109	B9	C119	A7	B10	D10	A113	D112
Allele dropout	0	1e−6	0	0	0.0074	4e−6	0	1e−6	0	0.0072	1e−6
False alleles	0	0	0	1e−6	0	0	1e−6	0	0.0122	0	1e−6

## APPENDIX D

**TABLE D1:** Parameter estimates from mixed effects models fit to composite resistance surfaces using data for 2018 and 1990.

Year	Resistance surface	Layers	Parameter	*β*	SE	*t*‐value
2018	Composite 1	Agr_dry; Agr_nat; Agr_tree; Forest; Slope; Urban	Intercept	.654	0.003	214.261
			Composite 1	0.008	0.001	13.737
	Composite 2	Agr_dry; Agr_nat; Agr_tree; Forest; Urban	Intercept	.654	0.003	214.457
			Composite 2	.007	0.001	13.764
	Composite 3	Agr_dry; Agr_nat; Agr_tree; Forest	Intercept	.654	0.003	214.060
			Composite 3	.008	0.001	13.707
	Composite 4	Agr_dry; Agr_tree; Forest	Intercept	.654	0.003	214.295
			Composite 4	.008	0.001	13.742
	Composite 5*	Agr_dry; Agr_tree	Intercept	.654	0.003	213.861
			Composite 5	.008	0.001	13.717
1990	Composite1	Agr_dry; Agr_tree; Forest; Pasture; Shrub_other; Shrub_scle; Slope	Intercept	.654	0.003	214.658
			Composite 1	.007	0.001	14.057
	Composite 2	Agr_dry; Agr_tree; Forest; Pasture; Shrub_other; Shrub_scle	Intercept	.654	0.003	214.345
			Composite 2	.007	0.001	14.028
	Composite 3	Agr_dry; Agr_tree; Forest; Shrub_other; Shrub_scle	Intercept	.654	0.003	214.709
			Composite 3	.007	0.000	14.045
	Composite 4	Agr_dry; Agr_tree; Forest; Shrub_scle	Intercept	.654	0.003	214.696
			Composite 4	.007	0.000	14.134
	Composite 5	Agr_dry; Agr_tree; Shrub_scle	Intercept	.654	0.003	214.655
			Composite 5	.007	0.000	14.133
	Composite 6	Agr_dry; Shrub_scle	Intercept	.654	0.003	214.291
			Composite 6	.007	0.000	14.025

*Note*: Beta, standard error (SE) and *t*‐value estimates are from model fit by restricted maximum likelihood. Models that performed better than distance alone are represented with an asterisk.

**TABLE D2:** Parameter estimates from the mixed effects models fit to optimized single resistance surfaces based on data for 2018 and 1990.

Year	Resistance surface	Parameter	*β*	SE	*t*‐value
2018	Agr_dry*	Intercept	.654	0.003	213.651
		agr_dry	.007	0.001	13.488
	Agr_nat*	Intercept	.654	0.003	214.721
		agr_nat	.006	0.000	13.178
	Agr_tree*	Intercept	.654	0.003	214.745
		agr_tree	.007	0.001	13.360
	Geographic distance	Intercept	.654	0.003	214.736
		Distance	.004	0.000	12.646
	Elevation	Intercept	.654	0.003	214.815
		elevation	.004	0.000	12.721
	Forest*	Intercept	.654	0.003	214.858
		forest	.005	0.000	13.097
	Pasture	Intercept	.654	0.003	214.717
		pasture	.004	0.000	12.743
	Shrub_other	Intercept	.654	0.003	214.618
		shrub_other	.004	0.000	12.770
	Shrub_scle*	Intercept	.654	0.003	214.178
		shrub_scle	.005	0.000	12.830
	Slope*	Intercept	.654	0.003	215.215
		slope	.005	0.000	13.199
	Urban*	Intercept	.654	0.003	214.863
		urban	.005	0.000	13.034
1990	Agr_dry*	Intercept	.654	0.003	214.462
		Agr_dry	.007	0.001	14.000
	Agr_nat	Intercept	.654	0.003	213.636
		Agr_nat	.005	0.000	13.278
	Agr_tree*	Intercept	.654	0.003	214.823
		Agr_tree	.006	0.000	13.496
	Geographic distance	Intercept	.654	0.003	214.762
		Distance	.004	0.000	13.086
	Elevation	Intercept	.654	0.003	214.907
		elevation	.004	0.000	13.210
	Forest*	Intercept	.654	0.003	214.597
		forest	.005	0.000	13.684
	Pasture*	Intercept	.654	0.003	214.235
		pasture	.008	0.001	13.724
	Shrub_other*	Intercept	.654	0.003	214.248
		shrubother	.006	0.000	13.602
	Shrub_scle*	Intercept	.654	0.003	214.772
		shrubscle	.005	0.000	13.452
	Slope*	Intercept	.654	0.003	214.850
		slope	.005	0.000	13.621

*Note*: Beta, standard error (SE) and *t*‐value estimates are from model fit by restricted maximum likelihood. Models that performed better than distance alone are represented with an asterisk.
